# How Do Machines Learn? Artificial Intelligence as a New Era in Medicine

**DOI:** 10.3390/jpm11010032

**Published:** 2021-01-07

**Authors:** Oliwia Koteluk, Adrian Wartecki, Sylwia Mazurek, Iga Kołodziejczak, Andrzej Mackiewicz

**Affiliations:** 1 Faculty of Medical Sciences, Chair of Medical Biotechnology, Poznan University of Medical Sciences, 61-701 Poznan, Poland; oliwiakoteluk@gmail.com (O.K.); a.wartecki25@gmail.com (A.W.); 2 Department of Cancer Immunology, Chair of Medical Biotechnology, Poznan University of Medical Sciences, 61-701 Poznan, Poland; a.mackiewicz@ump.edu.pl; 3 Department of Cancer Diagnostics and Immunology, Greater Poland Cancer Centre, 61-866 Poznan, Poland; 4 Postgraduate School of Molecular Medicine, Medical University of Warsaw, 02-091 Warsaw, Poland; kolodziejczak.iga@gmail.com

**Keywords:** machine learning, artificial intelligence, bioinformatics, medicine, algorithm, decision making, personalized medicine, data processing, data mining, personalized treatment

## Abstract

With an increased number of medical data generated every day, there is a strong need for reliable, automated evaluation tools. With high hopes and expectations, machine learning has the potential to revolutionize many fields of medicine, helping to make faster and more correct decisions and improving current standards of treatment. Today, machines can analyze, learn, communicate, and understand processed data and are used in health care increasingly. This review explains different models and the general process of machine learning and training the algorithms. Furthermore, it summarizes the most useful machine learning applications and tools in different branches of medicine and health care (radiology, pathology, pharmacology, infectious diseases, personalized decision making, and many others). The review also addresses the futuristic prospects and threats of applying artificial intelligence as an advanced, automated medicine tool.

## 1. Introduction

Living in the big data era, with billions of terabytes of data generated every year, it might be challenging for humans to proceed with all the information. However, Artificial Intelligence (AI) can lend a helping hand. In the past, machines have gained an advantage over humans in physical work, where automation contributed to industry and agriculture’s rapid development. Nowadays, machines are gaining an advantage over humans in typically human cognitive skills like analyzing and learning. Moreover, their communication and understanding skills are improving quickly. There are numerous examples where AI already achieves much better results than humans in analyzing [1,2,3].

The AI focuses on exploiting calculation techniques with advanced investigative and prognostic facilities to process all data types, which allows for decision-making and the mimicking of human intelligence. Such computational systems usually operate on large amounts of data and often integrate different types of input. AI is a broader field of science, and one of the most significant branches of AI in medicine is machine learning (ML). ML means understanding and processing information from a given dataset by the algorithm, namely machine. The word “learning” stands here as the machine’s ability to become more effective with training experience. Such a machine can quickly draw novel conclusions from the data that may be omitted by humans. Machines’ potential increases year by year, making them more autonomous. However, human interference is necessary and still has the final word about taking or not particular actions. At least for now. Will it change in the future? Will we let the AI perform actions itself, or will it remain only as a human tool? One thing is unquestionable—we must start accustoming ourselves to live alongside the machines that begin to equal or even surpass people in the processes of analyzing and deciding.

## 2. How Do Machines Learn

The machine learning process is very similar to the learning mechanisms and biochemical principles of the human brain. All the decisions a human makes result from billions of neurons that analyze images, sounds, smells, structures, and movements, recognize patterns, and continuously calculate probabilities and options. Machines can also analyze and calculate similar data, including smell sensing by the electronic nose [4].

### 2.1. The Main Components of the Machine Learning Process

ML algorithms are methods to perform calculations and predictions [5]. They require *inputs* (see Table 1. Glossary)—all data presented to the ML algorithm for analysis, e.g., patients’ genome sequencing data. The ML algorithm’s outcome is called the *output*; for instance, prediction of a patients’ susceptibility to cancer. The simple analysis usually does not require large amounts of data for obtaining a high accuracy prognosis. In the more advanced analysis, more input is required [6,7,8]. Although the relationship between inputs and outputs is more complex than this, generally, setting more inputs should provide more accurate outcomes.

In comparison to ML, AI acts in response to the environment to meet the defined goals. According to Turing’s test, AI must be contagious, embody its memory, be able to conclude, and adapt to new circumstances [9]. A good example is SIRI or ALEXA, where the AI performs different tasks such as voice recognition, number dialing, information searching to fulfill user’s requests [10,11]. AI gives a machine cognitive ability and therefore is more complicated than ML [12].

### 2.2. Machine Learning Models

**Figure 1:**
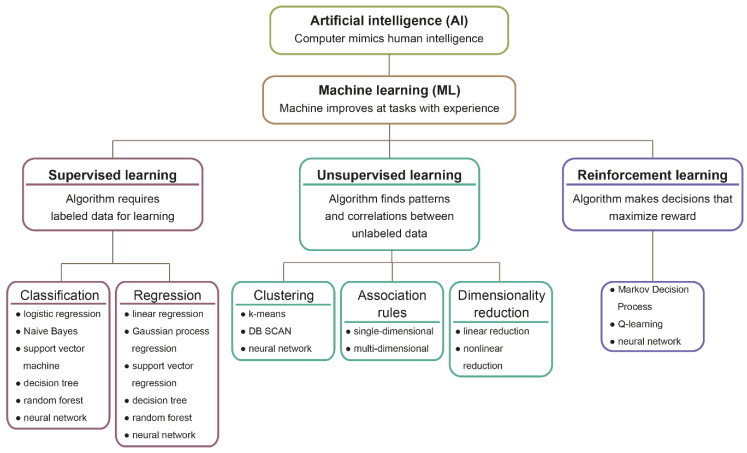
Machine learning models and algorithms. Machine learning is a subfield of artificial intelligence science that enables the machine to become more effective with training experience. Three principal learning models are supervised learning, unsupervised learning, and reinforcement learning. Learning models differ depending on the input data type and require various algorithms.

There are three principal learning models of the ML: supervised learning, unsupervised learning, and reinforcement learning, which differ depending on the type of data input. Different learning schemes require specific algorithms (Figure 1, Table 2).

**Figure 2:**
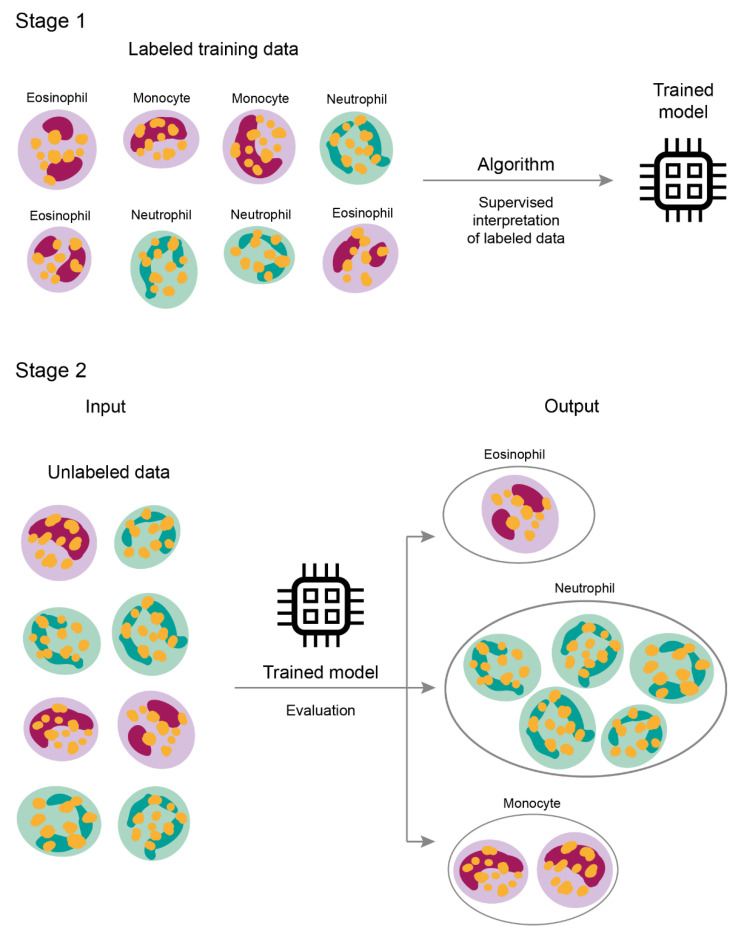
Supervised learning model. In the supervised method, learning begins on a labeled dataset, where input data is linked to its output label. The algorithm is then validated on a different, non-labeled dataset, not presented to the machine previously.

The supervised model requires the described data for learning. Hence an input with extracted *features* is linked to its output *label* (Figure 2) [22]. Therefore, after training, the algorithm can make predictions on non-labeled data. The output is generated by data classification or value prediction (Figure 1, Table 2). The classification bases on assigning elements into groups, having previously defined features, whereas the value is predicted based on training data calculations [15].

**Figure 3:**
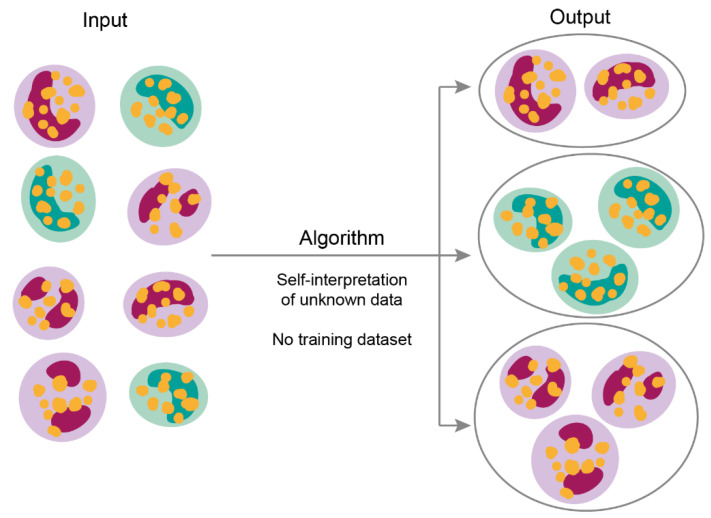
Unsupervised learning model. The algorithm extract features itself from the unknown input data without training. Hence the algorithm can cluster data with a similar component, which differentiates data from other objects or groups.

Contrarily, in unsupervised learning, the machine tries to find patterns and correlations between presented in randomized order examples that are not labeled, categorized, or classified. (Figure 3) [23]. The main unsupervised data mining methods are *clustering,* association rules, and dimensionality reduction (DR) [24,25,26]. The difference between clustering and classification is that grouping does not base on predefined features. Consequently, an algorithm must assemble data by characteristics, which differentiates them from other groups of objects. Besides data clustering, unsupervised learning allows detecting anomalies, meaning identify thighs that outline the other data points and differ from them [27].

The association rules mining is aimed to find common features and dependencies in a large dataset [26]. For example, Scicluna et al. classified the patients’ sepsis basing on the association of its endotypes to leukocyte counts and differentials [28]. This study allowed to predict a patient’s prognosis and mortality by characterizing blood leucocyte genome-wide expression profiles. Such classification would allow the identification of patient endotypes in clinical practice.

All of the data types ranging from MRI scans to digital photographs or speech signals usually are characterized by high dimensionality [29]. The data dimensions denote the number of features measured for every single observation. DR decreases the number of data features by selecting important attributes or combining traits. Concerning unsupervised learning, DR is used to improve algorithm performance, mainly by employing bias/variance tradeoff and thus alleviating overfitting [30]. Post-genomic data can serve as a good model of DR. Those data are often high-dimensional, contain more variables than samples, have a high degree of noise, and may include many missing values. The use of unsupervised learning would reduce the number of dimensions (e.g., variables), limiting the data set to only those variables with, e.g., the highest variance [31].

DR is performed through two categories of methods: feature selection and feature extraction. Feature selection takes a subset of features from original data that are the most relevant for a specific issue [32]. Feature extraction removes redundant and irrelevant features from the original data set, resulting in more relevant data for analysis [33]. The major difference between these two methods is that feature selection chooses a subset of original traits, and feature extraction produces new features distinct from the original ones. A wide range of linear and nonlinear DR methods is used to displace excessive features [34].

One of the most broadly used unsupervised learning methods for DR of large-scale unlabeled data is principal component analysis (PCA) [35]. The PCA method’s main aim is to determine all uncorrelated features called principal components (PC). PCA can be used in various applications such as image and speech processing, robotic sensor data, visualization, exploratory data analysis, and a data preprocessing step before building models [33,35].

Besides supervised and unsupervised models, some models cannot be classified strictly into these categories. In the first one, semi-supervised learning labeled training set is supported by an immense amount of unlabeled data during the training process. The main goal of including the unlabeled data into the model is improving the classifier [36]. What is more, it has been shown that using semi-supervised models can improve the generalizability of risk prediction when compared to supervised ones [37]. Another approach, named self-supervised learning, generates supervisory signals automatically from the data itself [38]. It is achieved by presenting an unlabeled data set, hiding part of input signals from the model, and asking the algorithm to fill in the missing information [39]. Presented methods eliminate the often-occurring problem, which is the lack of an adequate amount of labeled data. They are especially useful when working with deep learning algorithms and are gaining more and more popularity.

**Figure 4:**
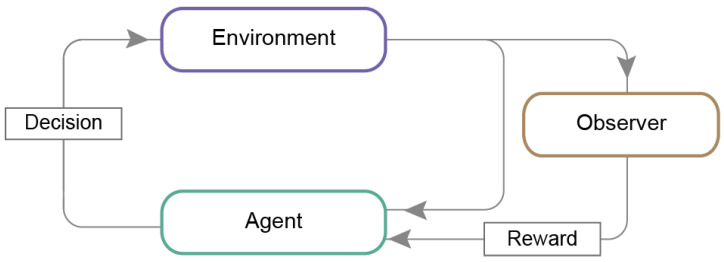
Reinforcement learning model. An agent in its current state performs an action, which influences the state of the environment. The environment gives back the information about its changed state to the agent. The supervisor interprets and rates the action, providing a reward for a correct decision.

In the reinforcement learning method, the algorithm learns by trial-and-error process, continually receiving feedback [40]. The *artificial agent* reacts to its *environment* signals representing the environment’s state (Figure 4). The actions performed by the agent influences the state of the environment. The foremost goal is to make decisions that guarantee the maximum *reward*. When the machine makes a correct decision, the supervisor gives a reward for the last taken action in the form of an assessment, for example, 1 for proper action and 0 for incorrect. However, when the machine chooses the next step erroneously, it is penalized [41]. The functional expression of reinforcement learning is a chess game, where an agent has to react to an opponent’s moves to get a maximal reward for its movement and win [42].

### 2.3. Deep Learning

Artificial neural networks (ANNs) are a subset of ML, where the model consists of numerous *layers*—functions connected just like neurons and acting parallel. ANNs that contain more than one hidden layer are thus referred to as “deep” [43]. Deep learning (DL) is built on interlinked multi-level algorithms, creating neural-like networks [6]. In other words, DL is a collection of complex functions that automatically discover relationships in raw data. Such a set is created by extracting higher abstraction from the data [44]. DL can also be categorized into supervised, semi-supervised, unsupervised, as well as reinforcement learning [45].

**Figure 5:**
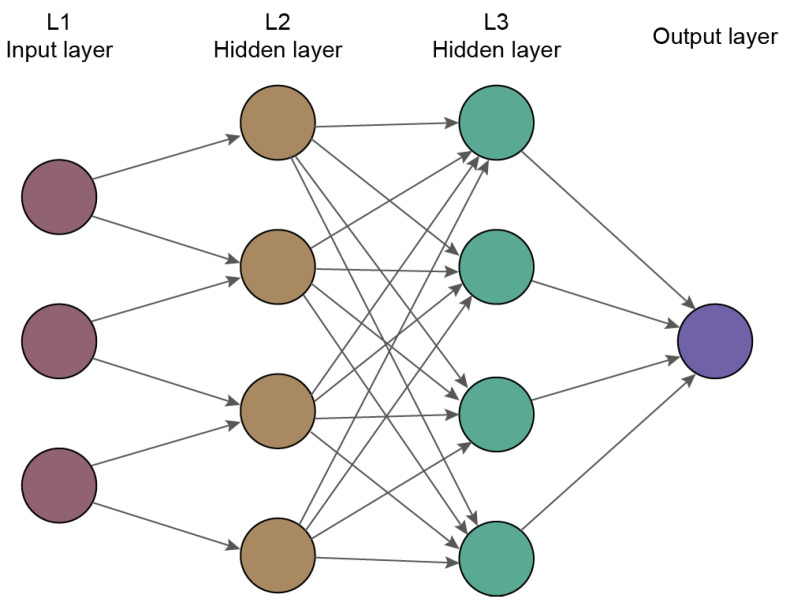
Layers of deep feedforward or feedforward neural network. The statement feedforward means that the information flows from input through some estimation functions to generate the output. Elsewhere exists a feedback neural network, where the information about the output is fed back to the model. Each layer represents a different function—the input layer (L1) with the first function, the hidden layers (L2, L3, …, LX) with the next functions. The depth of the model reflects the connection chain length. In the last output layer, the output value should match the input value approximated by earlier functions.

The main advantage of this method is that DL is capable of feature extraction with no human intervention. DL exploits a structure imitating a human’s neuronal structure of the brain (Figure 5). The structure consists of one input layer, some hidden layers, and one output layer wherein neurons (also nodes) are connected with other layers’ neurons. These connections are assigned a weight, which is calculated during the training process. The algorithm has to determine the best approximate output at each layer to get the desired final result [40,44,46].

The most straightforward neural network is called feedforward. The statement feedforward means that the information flows from input neurons through some estimation functions to generate the output. This DL operation provides no feedback between layers. Despite that, the backpropagation algorithm is often used with feedforward neural networks. It is a precise adjustment of neural network scales based on the error rate obtained in the previous training session. It allows for the calculation of the loss function gradient, including all the weights in the network. Proper weight tuning reduces the error level and increases the model’s reliability by increasing its generalization [47].

One of the most common deep neural networks is the convolutional neural network (CNN). It consists of the convolution, the activation layer, the pooling layer, and the fully-connected (classification) layer. The convolution layer comprises filters that extract and expand the number of features (parameters), represented as maps that characterize the input. The activation layer (which is mostly nonlinear) is composed of an activation function, and takes a generated map of features, and creates an activation map as its output. Then, the pooling layer is applied to reduce the spatial dimensions, hence achieving computational performance and lowering the likelihood of overfitting. Having processed the input by several such sets of layers, the classification occurs. The final output of CNN in the form of a vector serves as the input for the classification layer, where the algorithm produces the classifier, e.g., tumor or normal [40,45,47]. CNNs are used to analyze images, and in medicine, they are most helpful, for example, in radiology [40]. There are other various applications [48,49,50,51] described in Section 3, Application of Machine Learning in Medicine.

Although the simple CNN architecture may look like described, there are many variations and improvements. One of them is the fully convolutional network (FCN), which has convolutional layers instead of fully-connected layers. In opposite to CNN, FCN naturally handles inputs of any size and allows for pixel-wise prediction. In order to do this, FCN yields output with the input-like spatial dimensions. Such upsampling can be achieved by using deconvolution layers [52]. Therefore, the FCN is a well-suited option for semantic segmentation, especially in medical imaging. Ronneberger et al. created a U-Net that goes over 2D pictures [53]. The u-shape is created because of the upsampling part, where there are many feature channels, which allow the network to propagate context information to higher resolution layers. The U-net comprises of the contracting and expanding path. The convolution and the pooling layers in the contracting path extract advanced features and downsize the feature maps. Later the expansion path, consisted of the different convolution (“up-convolution”) and upsampling layers, restores the original map size. In addition to this, after each upsampling, the feature map from the same level of the contracting path is concatenated to give the feature localization’s information. At the final layer a 1 on 1 convolution is used to map each component feature vector to the desired number of classes. Similar but yet different is the V-net, presented by Milletari et al. [54]. In comparison to U-net, V-net learns a residual function at each stage and examines 3D pictures, using volumetric filters. Both networks performed outstandingly, being named a state-of-the-art in the medical image segmentation [55].

A noteworthy CNN variation is region-based CNN (R-CNN). R-CNN can find and classify any objects in an image by combining proposals of rectangular regions with CNN. R-CNN is an algorithm, which consists of two detection stages. The first stage identifies a subset of regions in the image that may contain an object, explicitly regions proposal. In the second stage, these regions are adequately classified by the CNN layers outputting classifier region of interest (ROI) and background [56]. This solution is successfully applied regarding tumor diagnosis from contours [57]. However, there are also more complex R-CNN subtypes. The fast R-CNN is more efficient owing to sharing the computations for overlapping regions. The fast R-CNN differs from R-CNN because, as input, it takes the entire image and a set of object proposals. Then, several convolutional and pooling layers produce the feature map. Given each ROI proposal, the pooling layer extracts a fixed-length feature vector from the feature map. All feature vectors are provided to a combination of fully connected layers and finally split into two output layers [58].

Additionally, there are implemented more advanced R-CNN. Instead of using an additional algorithm to generate proposal regions, the faster R-CNN uses proposal networks region. It is made up of convolutional layers and efficiently predicts region proposals, so the calculation is even faster [59]. Moreover, there is another R-CNN variant, namely Mask R-CNN. This complex method extends the faster R-CNN by adding a branch to predict segmentation masks for every ROI, together with the available branch of classification and regression of the bounding box regression. The mask’s branch is a minor FCN employed to individual ROI, forecasting the segmentation mask in a pixel-to-pixel manner [60].

Other interesting examples of DL methods are the recurrent neural network (RNN) and its variant long short-term memory network (LSTM). As distinct from the previously described neural network, RNN forms cycles in its structure. Such a network design enables recycling of its limited computational resources, thus performing more complex computations [61]. What is more, by using recurrent connections, a kind of memory is created so that RNN can learn from the information processed so far [62]. However, RNN may face the vanishing gradient problem encountered, e.g., during backpropagation [63]. Thus, variations of RNN were created, like LSTM. In LSTM, the recurrent hidden layer of RNN is replaced by a memory cell. It enables better reproduction of long-time dependencies [62].

### 2.4. Machine Learning Process

**Figure 6:**
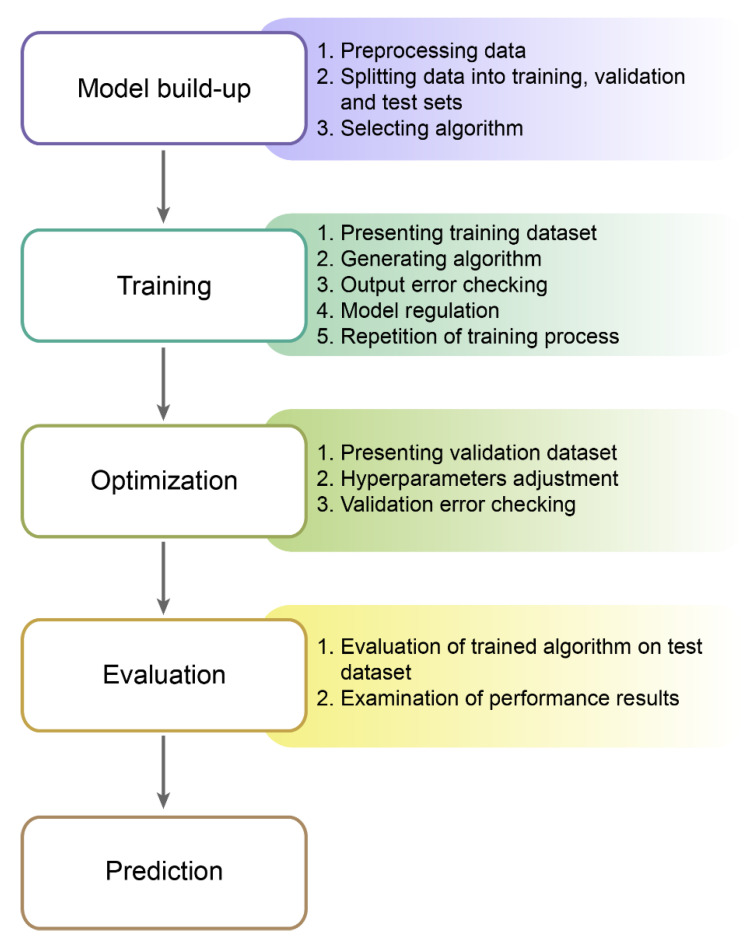
Machine learning process. The machine learning process starts with model build-up. Data need to be preprocessed and split into training, validation, and test sets in this step. The next stage is the training phase, during which parameters are adjusted on the training dataset. Then, during the optimization phase, hyperparameters are tuned on the validation dataset. After the last model adjustments, the trained algorithm processes the final test dataset, and the model performance results are examined.

The very first step of the learning process is data preparation (Figure 6). When working on big datasets, data will likely be unclean, i.e., incomplete, inconsistent, or corrupt. A better algorithm-based analysis requires a high-quality dataset without any anomalies or duplicates [64]. A good practice is to randomize inputs in order to exclude the influence of order on learning.

What is more, it is best to split data into three sets: training data, validation data, and test data [65]. This technique is termed the “lock box approach” and is a very effective practice in the learning process, commonly used in neuroscience [66]. Different datasets allow tuning some *hyperparameters* on validation data before testing the algorithm on the other datasets [67].

After having the data processed, the next step is selecting the algorithm and the learning model. The most common learning model is the supervised one [64,68]. Sometimes, the choice of an appropriate algorithm and a learning scheme depends on the type of data, e.g., categorical or numerical, and what task it needs to be automated. The supervised learning requires labeled data. In the case of an insufficient quantity of labeled data, unsupervised learning, semi-supervised, or self-supervised learning may be used [36,37,38,39,69]. The accuracy, size of the training data set, training time, and the number of parameters and features need consideration when selecting the algorithm.

During a training phase, the algorithm proceeds the training data. The outcome has to match the previously marked output. When the mistake occurs, the model is corrected, and another iteration is tested [70].

The validation dataset is to determine the best tuning of hyperparameters during the optimization phase [6,65]. If the validation error is high, the supervisor presents more data to the algorithm and regulates parameters. Sometimes, building a whole new model might be required. If the validation and training sets with the same normal distribution perform well, then it is likely for our machine learning model to perform effectively also on the test set [71].

The final phase is applying a test set to the trained model and checking the performance results. A test set must contain data instances not presented to the algorithm in the training and optimization phase [65,66]. Testing the model on the previously applied data can result in obtaining inflated performance scores [67].

### 2.5. Examples of Machine Learning in Everyday Life

ML is a universal tool, applied in many various fields, and often we are not even aware we use it daily. In the cyber-security sector, ML is used to protect the user, whereas it becomes more resilient with every known threat. A Ravelin company can detect fraud using the ML algorithm, which continually analyzes normal customer behavior [72]. When it spots suspicious signals, like copying and pasting information by resizing the window, the algorithm can block the transaction or flag it to review [73].

The AI, such as IBM Watson, is trained on billions of data artifacts from different sources like blogs. Afterward, AI concludes the relationship between threats such as malicious files or mistrustful IP, limiting time for analysis, and improving reaction to threat up to 60 times faster [3].

Considering the daily use, Netflix is an excellent example of a successful ML application. Behind their achievement stands personalization, where the platform, based on the user’s activity, recommends titles and visuals suited for them. Additionally, it helps the company to predict what content is worth investing [74].

A terrific example of the AI in ordinary routine is Waymo’s self-driven car, trained in 3D maps that point out information like road profiles, crosswalks, traffic lights, or stop signs [3,74,75]. The sensors and software scan around its neighborhood, and thus it can distinguish and predict traffic users’ movement based on their speed and trajectory [76].

## 3. Application of Machine Learning in Medicine

Techniques based on ML started to step into medicine in the 1970s, but over time, the possibilities for their use began to multiply [77,78]. The first-ever ML-based diagnostic system was already approved by the U.S. Food and Drug Administration (FDA) in 2018 [79]. The system implements “in silico clinical trials”, which helps develop more efficient clinical trial strategies. It allows investigators to detect safety and effectiveness signals earlier in the new drug development process and contributes to costs reduction [80].

With many hopes and expectations, ML has the capacity to revolutionize many fields of medicine, helping to make faster and more correct decisions and improving current standards of treatment. The potential applications of ML in general medicine are summarised in Table 3.

### 3.1. Imaging in Medicine

With an increased number of images taken every day, e.g., magnetic resonance imaging (MRI), computer tomography (CT), or X-rays, there is a strong need for a reliable, automated image evaluation tool. An interesting example is a tool created by Kermany et al., which, when adequately trained, has the potential of numerous applications in medical imaging [82]. It uses a neural network to analyze optical coherence tomography (OCT) images of the retina, allowing to diagnose macular degeneration or diabetic retinopathy with high accuracy and sensitivity. Moreover, this model could also indicate the cause of bacterial or viral pediatric pneumonia, making it a universal radiological tool. ML also allows creating images with better quality. In reconstructing the noisy image, the automated transform by manifold approximation (AUTOMAP) framework is used to obtain better resolution and quality [81]. As more details can be recognized, the diagnosis can be faster and more accurate.

The accuracy of imaging and its assessment is essential, especially in (the case of) detecting and diagnosing abnormalities in the development of the fetus. Parental diagnosis of fetal abnormalities has markedly benefited from the advances in ML. ML algorithms have been widely used to predict the risk of chromosomal abnormalities (i.e., euploidy, trisomy 21) or preterm births. The latest technological advances in ML also improve the diagnosis of fetal acidemia or hypoxia based on CTG analysis [115].

ML also progresses in imaging methods. In silico staining technique provides an excellent solution to microscopy problems, such as the need for additional staining to visualize some cells or tissue structures [83]. Based on patterns invisible to the human eye, the algorithm can accurately predict the cell nuclei’s location and size, cell viability, or recognize neurons among mixed cell populations.

Recent advances and using DL-based techniques enabled to read more information from various images. It is now possible to improve the transplantation process by using CNN [49]. The approach created by Altini et al. analyzes kidney histological slides and determines the global glomerulosclerosis (ratio between sclerotic glomeruli and an overall number of glomeruli), which is one of the necessary steps in the pre-transplantation process. By using DL, it can be assessed faster and with high accuracy, and therefore has the potential to quicken the whole transplantation process. Using automatic semantic segmentation of patients with autosomal dominant polycystic kidney disease enables noninvasive disease monitoring [48]. The introduction of the latest ML techniques also enables predicting less obvious information from microscopic section images. Two interesting examples determine RNA expression [50] and predict patient survival after tumor resection [51]. Schmauch et al. created the HE2RNA model, which correctly predicted transcriptome of different cancer types, detected molecular and cellular modifications within cancer cells, and was able to spatialize differentially expressed genes specifically by T cells or B cells [50]. A different study developed two CNN models that could predict survival from histological slides after the surgical resection of hepatocellular carcinoma. Both models outperformed a composite score incorporating all baseline variables associated with survival [51].

### 3.2. Personalized Decision Making

Fast and personalized decisions are crucial in almost every field of medical sciences. Moreover, detecting and predicting life-threatening conditions before their full clinical manifestation is a highly significant issue. Cardiology’s main goals focus on developing tools predicting cardiovascular disease risk [86] and the mortality rate in heart failure patients [87]. AI can also be applied for prognosis in acute kidney injury [84]. Physicians can be informed about the injury before changes, detectable with current methods, occur. AI uses a recurrent neural network trained with big datasets of over 700,000 adult patients. It can predict kidney function deterioration up to 48 h in advance, giving some extra time to improve patients’ condition.

The auspicious direction of AI is an individualized prediction of genetic disease occurrence based on the patient’s genome screening. Integrating genomic data with parameters such as lifestyle or previous conditions established the tool, which may be used in the early screening of abdominal aortic aneurysm [85].

Another useful tool, created for personalized nutrition, processes data (e.g., blood tests, gut microbiome profile, physical activity, or dietary habits) and predicts postprandial blood glucose level [88]. The evaluation indicated a high correlation between predicted and measured glycemic response, indicating high fidelity of ML application. Such an approach may be the beginning of the personalized nutrition era to program diet in other metabolic disorders.

As data suggest, the microbiome is strictly related to cancer, affecting tumorigenesis’s natural course. Specific microbial signatures promote cancer development and affect many aspects of cancer therapies, such as the treatment’s efficacy or safety. Hence, ML-driven gut microbiota analysis seems to be extremely useful in oncology to prevent cancer development, make an appropriate diagnosis, and finally treat cancer [111].

Early diagnosis is a crucial but often also challenging task. Here once again, ML proves useful. It is now possible to detect abnormalities in patients’ handwriting. Using ANN, the algorithm can determine whether a person may be affected by Parkinson’s Disease or how much the disease has already developed [97]. Often the symptoms of a particular condition are subtle and therefore difficult to observe. That is what happens with blepharospasm, which is caused by orbicularis oculi muscle contractions and, in most problematic cases, may result in complete closure of the eyelids and blindness. Based on ANN, AI software was created to deal with diagnosis making [98]. It analyzes recorded videos, recognizes facial landmarks, and can detect even subtle blinks and around the eye area movement, which are necessary for diagnosing this dystonia.

### 3.3. Drug Design

The traditional approach of new drug design is based on numerous wet-lab experiments and is costly and time-consuming. Solutions to these problems are combining traditional synthesis methods with ML techniques [90]. Granda et al. applied the algorithm to analyze obtained data and classify reagents as reactive or non-reactive, faster, and with high precision. The used approach is the beginning of creating an automated tool for chemicals discovery, contributing to new therapeutic compounds development. Screening big datasets of compounds to find ligands with target proteins is a very long part of the drug design process, even with utilizing ML. The fast-screening compounds tool, which uses traditional support vector ML and a graphics processing unit (GPU), was created to face this challenge. A GPU divides all the data into small parts and analyzes them simultaneously in smaller subsets, shortened screening time. The multi-GPU computers might reduce this time even more [91]. Applying a deep neural network enabled to screen of over 107 million molecules and identified a new antibiotic [92]. This compound, named halicin, differs in structure from previously known antibiotics and exhibits broad-spectrum activity in a mouse model, including pan-resistant bacteria.

Another big problem in the field of pharmacology is to identify the compounds’ mechanism of action. Yang et al. proposed a “white-box” ML approach that could identify new drugs and antibiotics mechanisms of action, contribute to overcoming antibiotic resistance, and design new therapeutics [89].

### 3.4. Infectious Diseases

Almost all global media in the first part of 2020 were dominated by information about the SARS-CoV2 outbreak. With a promptly increasing number of cases and COVID-19-related deaths, there is a strong need for tools to fast diagnoses, estimate epidemic trends, and determine viruses’ evolutionary history. Taking all of these needs into account, ML comes in handy. Combined ML techniques, such as neural network, support vector machine, random forest, and multilayer perception, were used to create a tool for rapid, early detection of SARS-CoV2 patients. This algorithm analyzes computed tomography (CT) chest scans and clinical information such as leucocyte count, symptomatology, age, sex and travel, and exposure history [104].

ML techniques are also beneficial for virologists and epidemiologists. Supervised learning with digital processing was used for the rapid classification of novel pathogens [103]. The authors created an alignment-free tool, which analyzes viral genomic sequences and enables tracking the evolutionary history of viruses and detecting their origin. Modeling epidemic trends is significant from the public health and health care system’s point of view. A combination of ML algorithms and mathematical models can reliably predict the number of confirmed cases, deaths, and recoveries in the peak of an epidemic several months earlier. What is more, it can estimate the number of additional hospitalizations, which gives the hospitals and health care facilities time to prepare [102].

## 4. Challenges and Prospects

It may seem that the revolution in biotechnology and information technology enables us to apply these fields in a very advanced way in everyday life. In a sense, this is true, but we must be aware that this revolution is just beginning and will move faster and faster.

We must not forget that machines have a significant advantage over humans in addition to being on par with human cognitive skills: they can be networked. How is this beneficial? Take an “AI doctor” as an example. Networked AI doctors could easily and rapidly exchange information, be actualized, and learn from each other. In contrast, it is impossible to actualize the knowledge of every single human doctor in the world. Furthermore, sometimes this knowledge might be life-saving information, for example, newly discovered symptoms and treatment of rapidly spreading disease, like COVID-19. Therefore, networked AI doctors’ abilities can be as valuable as numerous experienced human doctors of different specializations.

Some people fear that one mistake of the networked AI doctor could result in fatal consequences for thousands of patients worldwide within a few minutes. However, connected AI doctors could make their own independent decisions, considering the other AI doctors’ opinions. For example, a single patient living in a small village in Siberia or Tibet could benefit from comparing diagnosis coming from a thousand AI doctors [116,117,118]. AI could provide more accurate, faster, and cheaper health care for everyone. This vision is very futuristic but possible.

For now, ML enables human doctors to save their time, hospitals to save money, and patients to receive highly personalized and more accurate treatment. However, the progressing implementation of ML in medicine has many technical and ethical limitations. The main technical issue that ML needs to overcome is the number of potential manipulations of input data that can influence the system’s decisions. For example, a simple action as adding a few extra pixels or rotating the image can lead to misdiagnosing and cancer misclassification as malignant or benign [79]. Researchers worldwide are trying to find a way to trick trained ML models in various ways and improve them [119]. ML models’ good performance is strongly connected with the amount of data used in the training process—the larger the dataset, the better the model is trained. This creates a need to have a significant amount of good-quality data, which is not always easily accessible. On the other hand, knowing the weaknesses of AI creates a field for hackers to control it and influence its outcomes. Fortunately, machines do not yet make essential decisions that may affect human health or even life without human supervision.

ML’s introduction to health care requires many ethical and legal issues to be solved [120]. There are reasoned concerns that AI may mimic human biases and have a propensity for any kind of discrimination. However, machines would mimic human prejudice and favoritism only if the creator incorporates them into the algorithm. Another significant threat is the uncontrolled creation of algorithms to perform in an unethical way. Private IT companies, which want to produce medicine systems, will have to balance their profits and patients’ well-being. Given the above risks, it will be necessary for governmental authorities to create a legal practice of ML-based systems approval and precautions to identify potential mistakes, biases, and abuse.

Nowadays, when AI is all around, and humans interact with it on a regular basis, we may perceive the mind in the machines. Recent results suggest that most people report various emotions when interfacing with a system using ML [121]. The majority of people feel surprised or amazed by AI’s extraordinary outputs and its anthropomorphic qualities. However, AI-based systems also can arouse negative emotions such as discomfort, disappointment, confusion, or even fear. One thing is sure, ML models used in health care will need to earn patients’ and doctors’ trust.

Some may argue that we will never let machines make their own decisions, but we already did in many fields. What is more alarming, many of us do not even know about it. Popular music applications decide what songs or artists they should recommend to us to match our taste or how often we need a random surprise to make us satisfied with the application. Everything we liked, watched, how many times went back to see the same picture, and how much time we spent on particular pictures is analyzed by social media algorithms. Based on all the gathered information, the algorithms recommend movies, posts, friends, advertisements. Moreover, the algorithms analyze us in terms of the likelihood of joining a particular group or organization. This sounds scary, but actions performed by machines are already influencing our decisions and lives daily.

There is no doubt that, if ethically and adequately trained, ML improves medicine and health care. Nevertheless, it also leaves many unanswered questions. Should physicians have better knowledge about the construction and limitations of these tools? Are we able to trust the machines with our health and life? Will we allow the machines to think entirely by themselves? Will algorithms still require medical or bioinformatical supervision? Who will bear the blame for ML mistakes? For now, we are sure that artificial intelligence is not only the future but also the present.

## Figures and Tables

**Table 1:** Glossary.

Element of ML	Description
Artificial agent	An independent program that acts regarding received signals from its environment to meet designated goals. Such an agent is autonomous because it can perform without human or any other system.
Clustering	Grouping data points with similar features which differ from other data points containing exceedingly different properties.
Environment	A task or a problem that needs to be resolved by the agent. The environment interacts with the agent by executing each received action, sending its current state and reward, linked with agents’ undertaken actions.
Feature	An individual quantifiable attribute for the presented event, as the input color or size.
Hyperparameters	Parameters that cannot be estimated from training data and are optimized beyond the model. They can be tuned manually in order to get the best possible results.
Input	A piece of information or data provided to the machine in pictures, numbers, text, sounds, or other types.
Label	A description of the input or output; for example, an x-ray of lungs may have the label “lung”.
Layer	The most prominent structure in deep learning. Each layer consists of nodes called neurons, connected, creating together a neural network. The connections between neurons are weighted, and in consequence, the processing signal is increased or decreased.
Output	Predicted data generated by a machine learning model with an association with the further given input.
Reward	Information from the environment (or supervisor) to an agent about the action’s precision. The reward can be positive or negative, depending on if the action was correct or not. It allows concluding behavior in a particular state.

**Table 2:** Examples of specific algorithms suited for different learning models.

Algorithm	Prediction	References
**Algorithms Applied in Supervised Learning**		
Probabilistic model (classification)	Probability distributions are applied to represent all uncertain, unnoticed quantities (including structural, parametric, and noise-related aspects) and their relation to current data.	[13]
Logistic regression (classification)	Predicts probability comparing to a logit function or decision trees, where the algorithm divides data according to the essential assets making these groups extremely distinct.	[14]
Naïve Bayes classifier (classification)	Assumes that a feature presence in a class is unrelated to any other element’s presence.	[15]
Support vector machine (classification)	The algorithm finds the hyperplane with the immense distance of points from both classes.	[16]
Simple linear regression (value prediction)	Estimates the relationship of one independent to one dependent variable using a straight line.	[17]
Multiple linear regression (value prediction)	Estimates the relationship of at least two independents to one dependent variable using a hyperplane.	[18]
Polynomial regression (value prediction)	Kind of linear regression in which the relationship between the independent and dependent variables is projected as an n-degree polynomial.	[19]
Decision-tree (classification or value prediction)	A non-parametric algorithm constructs classification or regression in the form of a tree structure. It splits a data set into further small subsets while gradually expanding the associated decision tree.	[15]
Random forest (classification or value prediction)	Set consisting of a few decision trees, out of which a class dominant (classification) or expected average (regression) of individual trees is determined.	[20]
**Algorithms Applied in Supervised Learning**		
K-means (clustering)	Clusters are formed by the proximity of data points to the middle of cluster—the most minimal distance of data points in the center.	[21]
DBSCAN (clustering)	Consists of clustering points within nearby neighbors (high-density region), outlying those being comparatively far away (low-density region).	[21]
**Algorithms Applied in Reinforcement Learning**		
Markov Decision Process	A mathematical approach where sets of states and **rewards** are finite. The probability of movement into a new state is influenced by the previous one and the selected action. The likelihood of the transition from state “a” to state “b” is defined with a reward from taking particular action.	[21]
Q-learning	Discovers an optimum policy and maximizes reward for the whole following steps launched from the present state. Hither, the agent acts randomly, exploring and discovering new states or exploiting provided information on the possibility of initiating action in the current state.	[20]

**Table 3:** Potential applications of machine learning in medicine.

Branch of Medicine	Application	Description	ML Method	References
Radiology	Image reconstruction	High resolution and quality images	Deep neural network	[81]
	Image analysis	Faster and more accurate analysis	Convolutional neural network and transfer learning	[82]
Pathology	in silico labeling	No need for cell/tissue staining; faster and cheaper analysis	Deep neural network	[83]
Nephrology	Prediction of organ injury	Detection of kidney injury up to 48 h in advance, which enable early treatment	Deep neural network	[84]
	Image analysis and diagnosis	Polycystic kidneys segmentation	Convolutional neural network	[48]
Cardiology	Personalized decision making	Early detection of abdominal aortic aneurysm	Agnostic learning	[85]
		Improvement of ML techniques to cardiovascular disease risk prediction	Principal component analysis and random forest	[86]
		Mortality risk prediction model in patients with a heart attack	Decision tree	[87]
Nutrition	Personalized decision making	More accurate, personalized postmeal glucose response prediction	Boosted decision tree	[88]
Diabetology				
Transplantology	Computer-Aided Diagnosis	Estimation of global glomerulosclerosis before kidney transplantation	Convolutional Neural Network	[49]
Pharmacology	Studying drug mechanisms of action	New mechanisms of antibiotic action	White-box machine learning	[89]
	Predicting compounds reactivity	Automated tool for reactivity screening	Supported vector machine	[90]
	Ligands screening	Faster screening of compounds that bind to the target	Supported vector machine	[91]
	Compounds screening	Discovery of new antibacterial molecules	Deep neural network	[92]
	*De novo* drug design	Generation of libraries of a novel, potentially therapeutical compounds with desired properties	Reinforcement neural network	[93]
Psychiatry	Image analysis and diagnosis	MRI image analysis and fast diagnoses of schizophrenia	Supported vector machine	[94]
Neurology	Image analysis and diagnosis	MRI image analysis and diagnoses of autism spectrum disorder	A naïve Bayes, supported vector machine, random forest, extremely randomized trees, adaptive boosting, gradient boosting with decision tree base, logistic regression, neural network	[95]
	Prognosis the course of the disease	Prediction of progression of disability of multiple sclerosis patients	Decision tree, logistic regression, supported vector machine	[96]
	Diagnosis support	Mild and moderate Parkinson’s Disease detection and rating	Artificial Neural Network	[97]
	Diagnosis support	Blepharospasm detection and rating	Artificial Neural Network	[98]
Dentistry	Personalized decision making	Determination of optimal bone age for orthodontal treatment	k-nearest neighbors, a naïve Bayes, decision tree, neural network, supported vector machine, random forest, logistic regression	[99]
Emergency medicine	Personalized decision making	Triage and prediction of septic shock in the emergency department	Supported vector machine, gradient- boosting machine, random forest, multivariate adaptive regression splines, least absolute shrinkage and selection operator, ridge regression	[100]
Surgery	Personalized decision making	Prediction of the amount of lost blood during surgery	Random forest	[101]
Infectious diseases	Estimation of epidemic trend	Prediction of number of confirmed cases, deaths, and recoveries during coronavirus outbreak	Neural network	[102]
	The evolutionary history of viruses	Classification of novel pathogens and determination of the origin of the viruses	Supervised learning with digital processing (MLDSP)	[103]
	Diagnoses of infectious diseases	Early diagnoses of COVID-19	Convolutional neural network, support vector machine, random forest, and multilayer perception	[104]
Oncology	Patients screening	Indicating increased risk of colorectal cancer, early cancer detection	Decision tree	[105,106]
	Cancer research	New cancer driver genes and mutations discovery	Random forest	[107,108]
	Cancer subtypes classification	three-level classification model of gliomas	Support vector machine, decision tree	[109]
	Image analysis and cancer diagnosis	Prediction of gene expression	Deep learning	[50]
	Improvement of image analysis	Tumor microenvironment components classification in colorectal cancer histological images	1-nearest neighbor, support vector machine, decision tree	[110]
	Cancer development preventing	Gut microbiota analysis in search of biomarkers of neoplasms	Convolutional neural network, support vector machine, random forest, and multilayer perception	[111]
	Tolerability of cancer therapies	Identification of microbial signatures affecting gastrointestinal drug toxicity	A naïve Bayes, supported vector machine, random forest, extremely randomized trees, adaptive boosting, gradient boosting with decision tree base, logistic regression, neural network	[111,112]
	Image analysis and prognosis the course of the disease	Predicting hepatocellular carcinoma patients’ survival after tumor resection based on histological slides	Deep learning	[51]
	Treatment response prediction	Prediction of therapy outcomes in EGFR variant-positive non-small cell lung cancer patients	Deep learning	[113]
	Image analysis	Tumor microenvironments components identification	Support vector machine	[114]

## Data Availability

No new data were created or analyzed in this study. Data sharing is not applicable to this article.
